# Decoding Lower Limb Muscle Activity and Kinematics from Cortical Neural Spike Trains during Monkey Performing Stand and Squat Movements

**DOI:** 10.3389/fnins.2017.00044

**Published:** 2017-02-07

**Authors:** Xuan Ma, Chaolin Ma, Jian Huang, Peng Zhang, Jiang Xu, Jiping He

**Affiliations:** ^1^Neural Interface and Rehabilitation Technology Research Center, School of Automation, Huazhong University of Science and TechnologyWuhan, China; ^2^Center for Neuropsychiatric Disorders, Institute of Life Science, Nanchang UniversityNanchang, China; ^3^Center for Neural Interface Design, School of Biological and Health Systems Engineering, Arizona State UniversityTempe, AZ, USA; ^4^Department of Rehabilitation Medicine, Tongji Hospital, Tongji Medical College, Huazhong University of Science and TechnologyWuhan, China; ^5^Collaborative Innovation Center for Brain Science, Huazhong University of Science and TechnologyWuhan, China; ^6^Advanced Innovation Center for Intelligent Robots and Systems, Beijing Institute of TechnologyBeijing, China

**Keywords:** lower limb motor control, primary motor cortex, cortical neuronal encoding, electromyography (EMG), Kalman filter, nonhuman primates

## Abstract

Extensive literatures have shown approaches for decoding upper limb kinematics or muscle activity using multichannel cortical spike recordings toward brain machine interface (BMI) applications. However, similar topics regarding lower limb remain relatively scarce. We previously reported a system for training monkeys to perform visually guided stand and squat tasks. The current study, as a follow-up extension, investigates whether lower limb kinematics and muscle activity characterized by electromyography (EMG) signals during monkey performing stand/squat movements can be accurately decoded from neural spike trains in primary motor cortex (M1). Two monkeys were used in this study. Subdermal intramuscular EMG electrodes were implanted to 8 right leg/thigh muscles. With ample data collected from neurons from a large brain area, we performed a spike triggered average (SpTA) analysis and got a series of density contours which revealed the spatial distributions of different muscle-innervating neurons corresponding to each given muscle. Based on the guidance of these results, we identified the locations optimal for chronic electrode implantation and subsequently carried on chronic neural data recordings. A recursive Bayesian estimation framework was proposed for decoding EMG signals together with kinematics from M1 spike trains. Two specific algorithms were implemented: a standard Kalman filter and an unscented Kalman filter. For the latter one, an artificial neural network was incorporated to deal with the nonlinearity in neural tuning. High correlation coefficient and signal to noise ratio between the predicted and the actual data were achieved for both EMG signals and kinematics on both monkeys. Higher decoding accuracy and faster convergence rate could be achieved with the unscented Kalman filter. These results demonstrate that lower limb EMG signals and kinematics during monkey stand/squat can be accurately decoded from a group of M1 neurons with the proposed algorithms. Our findings provide new insights for extending current BMI design concepts and techniques on upper limbs to lower limb circumstances. Brain controlled exoskeleton, prostheses or neuromuscular electrical stimulators for lower limbs are expected to be developed, which enables the subject to manipulate complex biomechatronic devices with mind in more harmonized manner.

## 1. Introduction

Brain machine interface (BMI), which translates the activity of cortical neurons into specific motor output commands, has been assumed as a promising approach to help patients who have lost normal motor functions due to spinal cord injury or other neural impairments to restore fundamental skills in daily living (Serruya et al., 2002; Andersen et al., 2004; Schwartz, 2004; Lebedev and Nicolelis, 2006; Hatsopoulos and Donoghue, 2009). Extensive literatures have shown successful realization of the concept of BMI on both nonhuman primates (NHP) and human subjects (Wessberg et al., 2000; Taylor et al., 2002; Carmena et al., 2003; Hochberg et al., 2006; Kim et al., 2008; Velliste et al., 2008; Hwang and Andersen, 2009; Carpaneto et al., 2011; Hochberg et al., 2012; Wodlinger et al., 2014). Among the existing BMI studies, the majority is concerned with using motor cortical activity to decode the movements of upper limbs. Previous literatures have shown two-dimensional or three-dimensional hand/arm trajectories when monkey performing cursor tracking, joystick playing, target-pursuit or center-out tasks can be extracted from neural spike trains using linear regression (Moran and Schwartz, 1999; Gao et al., 2003; Hatsopoulos et al., 2004; Kemere et al., 2004; Paninski et al., 2004; Linderman et al., 2008), artificial neural networks (ANN) (Fang et al., 2010; Huang et al., 2011; Wang et al., 2014), or different variants of Bayesian filtering algorithms (Wu et al., 2002; Brockwell et al., 2004; Wu et al., 2006; Li et al., 2009; Wu et al., 2009; Wang and Principe, 2010; Kang et al., 2015; Menz et al., 2015; Hotson et al., 2016). Analogous approaches were also proposed for predicting arm electromyograph (EMG) signals from the discharge activity of an ensemble of motor neurons or multi-channel local field potentials (Morrow and Miller, 2003; Pohlmeyer et al., 2007; Nazarpour et al., 2012; Shin et al., 2012; Liao et al., 2015). In fact, among the existing population of neural trauma victims, a large portion suffer from the inability of standing and walking (Qin et al., 2010). With the expectation of helping them, researchers have begun to seek possible realizations of BMI systems for lower limb motor function restoration. Some studies have tried to unscramble the information correlating with user intentions (Kilicarslan et al., 2013) or lower limb kinematics (Presacco et al., 2011, 2012; He et al., 2014; Luu et al., 2016) or surface EMG signals (Paek et al., 2013) during treadmill walking or sitting/standing (Bulea et al., 2014) from noninvasive scalp electroencephalographic (EEG) signals. To gain more specific information about how cortical neurons modulate their activities to initiate and control lower limb movements, it is expected to directly collect neural activity from the cerebral cortical areas related to lower limbs using invasive electrodes. However, there are relatively scarce reports about these due to the lack of proper experimental paradigms or apparatus and the difficulties in locating the exact functional cortical areas related to lower limb movements compared to the cases in upper limb. Nicolelis et al. (Fitzsimmons et al., 2009) extracted kinematic parameters for monkey bipedal walking from cortical neuronal ensemble activity and proposed possible approaches to control artificial actuators that reproduce walking patterns.

The construction of BMI lies on the detection of cortical neuronal representations of limb movements. Generally speaking, the control and organization of movements are often considered different for upper limb and lower limb (Winters and Woo, 2012). For example, bipedal locomotion of human is dynamically controlled by the skillfully coordinated activations of the redundant lower limb muscles. Under normal conditions, the activations of these muscles are controlled by both specialized neural circuits in spinal cord known as central pattern generator (CPG) and command signals directly from the brain (Ogihara and Yamazaki, 2001; Vogelstein et al., 2006). Here we concentrate a fundamental lower limb voluntary movement: stand and squat. We previously reported a system for training monkeys to perform visually guided stand and squat tasks (Ma and He, 2010). Neuronal ensemble activity from the representation area of the lower limbs in the primary motor cortex (M1), subdermal intramuscular EMG signals in lower limb muscles, and motion trajectories were recorded synchronously with the task. In an acute data recording session, we have detected many M1 neurons tuned to certain task events (Ma et al., 2015).

In this study, we immediately followed our previous work to investigate issues related to neural decoding toward BMI applications. To what extent do neurons in M1 participate the initiation or maintenance of lower limb voluntary movements? Are the neurons showing correlation with certain task events we have observed sufficient for decoding both lower limb muscular activity and kinematics? Which computational models are better for describing the neuronal representation of such visual guided stand/squat movements? We were also curious about whether analogous methods which have been proven practical in upper limb scenario as mentioned above could achieve the same performance in lower limb cases. With the guidance provided by the data collected in acute recording sessions, we identified those locations where more task related neurons clustered, and subsequently carried out chronic electrode array implantation. A recursive Bayesian estimation framework was proposed for decoding EMG signals together with kinematics from cortical spike trains. Two specific algorithms were implemented and compared: a standard Kalman filter and an unscented Kalman filter. For the latter one, an artificial neural network was incorporated to deal with the nonlinearity in neural tuning. Our results indicate that lower limb EMG signals and kinematics during monkey stand/squat can be accurately decoded from the activity of M1 neurons.

## 2. Materials and methods

### 2.1. Overview

The data used in this study were collected from two male Rhesus monkeys (monkey 1 and monkey 2). During earlier experimental stage, acute neural recordings were performed for locating the exact brain areas corresponding to the stand/squat movements. After that, chronic electrode array implantation and data recordings were carried out subsequently. In both acute and chronic data recording sessions, subdermal intramuscular EMG signals and limb trajectories were synchronously recorded. Details about animal behavioral training, surgery and some preliminary results of the acute neural data recordings can be found in our previous papers (Ma and He, 2010; Ma et al., 2015). The animal behavioral training and the actual experimental protocols was designed and implemented at Arizona State University (ASU). The experiments were complied with NIH policy on Humane Care and Use of laboratory animals, and were approved by the institutional Animal Care and Use Committee of ASU.

### 2.2. Behavioral tasks

**Figure 1:**
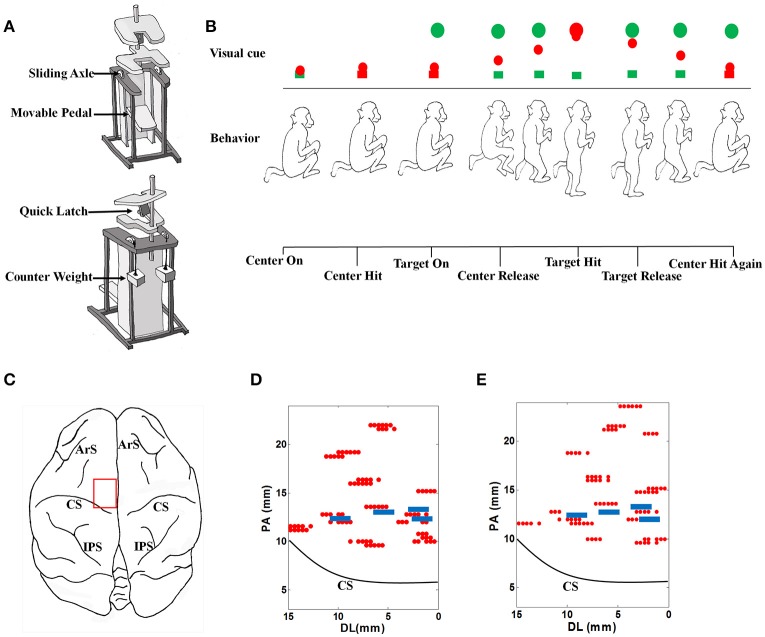
**The experimental setup. (A)** The specially designed primate chair for the stand/squat tasks. **(B)** The monkey experimental protocol and the definition of behavior events. **(C)** The target brain area for electrode implantation. ArS, Arcuate Sulcus; CS, Central Sulcus; IPS, Intraparietal Sulcus. **(D)** Locations of acute electrode penetrations and chronic electrodes for monkey 1. The red dots indicate the locations of acute electrode penetrations, and the 4 blue rectangles indicate the locations of the 4 chronically implanted microelectrode arrays. These locations are close to the midline (0 mm of the horizontal axis). PA, posterior- anterior. DL, dorsal- lateral. **(E)** Locations of acute electrode penetrations and chronic electrodes for monkey 2. The meanings of markers in the plot are the same as those in **(D)**.

The monkey performed stand/squat tasks following a series of visual cues in a virtual reality environment. Restrained in a special designed primate chair shown as Figure 1A, with the head and both arms fixed and both legs staying on the movable pedal, the monkey could stand upright by pushing down the movable pedal or squat down by contracting legs back. A marker for optical tracking was placed at the animal's right ankle, corresponding to a red ball in the virtual environment, which was defined as the ankle cursor.

The procedures of the behavioral task in a typical successful trial are shown in Figure 1B. The emerging of a green box at the bottom of the screen indicated the start of a trial (Center On). To proceed, the monkey must squat down and make the ankle cursor touch the green box (Center Hit). Once the box was touched, its color turned to red immediately. A short time later, a green ball (Target) emerged at the top of the screen (Target On). When perceiving this, the monkey should stand up and push down the pedal to move the ankle cursor toward the target until their positions were matched (Target Hit). The time point when the ankle cursor left the bottom box was defined as Center Release, at which the bottom box turned back to green. The monkey was trained to fully extend the legs to hit the target green ball and hold this standing posture up to about 400 ms. After that, it was required to squat by retracting the legs and make the ankle cursor move toward the starting position (Target Release). When the ankle cursor touched the bottom box again (Center Hit Again), the phase of squatting was finished. About 100 ms later, a trial was completed, and the monkey will receive several drops of water as the reward. Each trial was preceded by an inter-trial interval (varied randomly from 5 to 10 s) when the screen was illuminated with bright blank scene to prevent dark adaptation since the room was in low light condition.

### 2.3. Data collection and preprocessing

#### 2.3.1. Acute neural data recording

For acute neural data recordings, five independently controllable electrodes were transdurally insert into the target brain area (Figure 1C) in the left hemisphere with a multi-electrode micro-drive (Thomas Recording, Germany). Extra-cellular neural signals were amplified, filtered and recorded with a 64 -channel Plexon MAP system (Plexon Inc., Dallas, TX) at 40 kHz/channel sampling rate. Every 40 successful trials were grouped to form a recording dataset. When a dataset was finished, the locations or penetrating depth of the electrodes will be changed. The locations for electrode penetrating can be found in Figures 1D,E for each monkey. Putative neuron units were isolated through spike sorting based on the clustering of detected action potential waveforms in principal components (PC) feature spaces using the Offline Sorter (Plexon Inc., Dallas, TX) software. For each putative neuron, the coordinates corresponding to the electrode were recorded as its location. By calculating the spike counts in consecutive 30 ms nonoverlap bins, discrete spike times were transformed into a firing rate sequences for further analysis.

#### 2.3.2. EMG data collection

Separate surgical procedures were performed to implant Teflon coated stainless steel wire electrodes into 8 muscles on each leg, as listed below: right and left soleus (RS and LS), right and left tibialis anterior (RTA and LTA), right and left semitendinosus (RST and LST), right and left recutus femoris (RRF and LRF), right and left extensor digitorum longus (REDL and LEDL), right and left flexor digitorum longus (RFDL and LFDL), right and left adductor magnus (RMG and LMG), right and left flexor hallucis longus (RFHL and LFHL). The EMG signals were recorded at a sampling rate of 1 kHz. Only signals from the right leg were used in further analysis. Raw EMG signals were full-wave rectified and filtered with a 4-pole 10 Hz low-pass Butterworth filter to get nonnegative envelops.

#### 2.3.3. Kinematic data collection

The position of the ankle LED marker were captured by a VZ 2000 tracker (PTI, Canada) with a sampling interval of 15 ms and recorded synchronously with the neural data. The velocity of the ankle was approximated by calculating the difference between the position of the marker at current time and that at the previous time, while the acceleration from the velocity in the same way. Since the monkey was restrained on the primate chair when performing repetitive stand/squat behaviors, it is obvious that only the dimension corresponding to the axis perpendicular to the transverse plane (z axis) changed significantly in different movement stages. In fact, motion data on the other two dimensions remained almost invariant during the whole movement. Therefore, we defined the kinematics vector as the instantaneous position, velocity and acceleration of the ankle marker at the z axis. The EMG signals and kinematics were also down-sampled to match the bin size of the neural firing rate sequences.

#### 2.3.4. Identifying neural facilitation of muscle activation

During the acute data recording session, we have explored a wide region in M1. To determine the optimal locations for chronic electrode implantation, it was necessary to identify those areas which clustered more neurons with high predictive power on EMG signals or motion trajectories. Here we calculated the spike triggered average (SpTA) of each neuron-muscle pair to examine if a given M1 neuron facilitated certain lower limb muscle activation.

Previous studies have demonstrated how the SpTA of EMG signals can be used to identify the synaptic connectivity between cortical neurons and the motor units of activated limb muscles (Fetz and Cheney, 1980; McKiernan et al., 1998; Griffin et al., 2008). By taking into account post-spike characteristics of the averaged EMG such as the onset latency, peak magnitude and duration, SpTA can be used as a predictor of the quality and strength of facilitative contribution that a detected cortical neuron may have in relation to a given muscle (Baker and Lemon, 1998; Aguayo et al., 2009).

For each neuron-muscle pair, the SpTA was calculated by averaging full-wave rectified EMG segments for all successful trials starting 20 ms prior to until 60 ms after a spike event. For each SpTA, a baseline mean and standard deviation (SD) were calculated using the 20–10 ms of the SpTA prior to the spike event. The onset latency is defined as the point that the SpTA crosses a threshold defined by the baseline mean + 2 SD after the spiking events for the first time. Similarly, offset latency is defined as the point that the SpTA falls below a threshold (baseline mean − 2 SD). The peak magnitude is defined as the peak value of an SpTA after the spiking events minus the baseline mean. The peak width at half magnitude (PWHM) is defined as the width of the SpTA at a level that is half the measured peak. A neuron-muscle pair will be considered to have no dependency relationship and eliminated from statistics if the onset latency of the SpTA is less than 5 ms or greater than 15 ms, or the PWHM of the SpTA is less than 0.75 ms or greater than 9 ms.

After scanning enough datasets, a number of neurons which were considered to facilitate the activation of each given muscle were identified from the ensemble. The densities of different muscle-innervating neurons were calculated with regard to each muscle as the number of identified neurons divided by the total number of neurons recorded in an unit brain area. By plotting a series of density contours, the spatial distributions of these muscle-innervating neurons can be directly perceived and utilized to determine the locations for chronic implantation.

#### 2.3.5. Chronic neural data recording

About 1 month after the acute recording session, new surgeries were performed to implant 4 microelectrode arrays chronically into the selected brain areas for each monkey (Figures 1D,E). There were 16 polyimide insulated tungsten electrodes on each array as the recording channels, which were arranged with two rows. The diameter of each electrode was 50 microns. The spacing between electrodes in each row was 250 microns and the spacing between each row was 500 microns. In the following chronic neural data recording session, behavior tasks for monkey training and protocols for data recordings remained consistent.

### 2.4. Neural decoding algorithms

The purpose of the neural decoding algorithm is to use cortical spike trains to estimate the lower limb EMG signals together with the kinematics during the stand/squat tasks. We formulated the problem in a recursive Bayesian estimation framework (Bergman, 1999), where the activity of each muscle, characterized by corresponding EMG signals, or the motion of the limb, represented by the kinematics, were viewed as hidden states of a dynamic system, while cortical spiking data acquired by the implanted microelectrodes as constantly updated measurements to the system. In the following sections, we first introduce the general basics of the recursive Bayesian estimation theory we count on, and then describe the two specific algorithms for practical implementation in this study.

#### 2.4.1. Recursive bayesian estimation

Here **x**_*k*_ denotes the system states at the k-th time instant *t*_*k*_ = *k*Δ*t*. For EMG decoding, **x**_*k*_ is specified as ${\text{x}}_{k}={\left[u_{1}\left(k\right),u_{2}\left(k\right),\cdots \;,u_{M}\left(k\right)\right]}^{T}$, where *u*_*m*_(*k*), *m* = 1, ⋯ , *M* is the EMG envelop amplitude of the *m th* muscle at time *t*_*k*_, and *M* is the total number of muscles. For kinematics decoding, **x**_*k*_ is specified as ${\text{x}}_{k}={\left[z\left(k\right),v\left(k\right),a\left(k\right)\right]}^{T}$, representing the *z*-position, *z*-velocity and *z*-acceleration of the ankle marker at *t*_*k*_. Meanwhile, **y**_*k*_ denotes the measurement to the system at *t*_*k*_, which is in fact a vector containing the spike counts of *C* neurons in the *k th* time bin. The estimation consists of two steps: prediction and update. The aim is to construct the a-posterior probability density function *p*(**x**_*k*_|**y**_1:*k*_) for **x**_*k*_ conditioned on all available measurements up to *t*_*k*_ (**y**_1:*k*_).

In the prediction step, the a-priori distribution of **x**_*k*_ is estimated from the states at previous time instants. In terms of an usually made assumption that **x**_*k*_ is generated by a Markov process, we have

(1) $$\begin{array}{l} p\left({\text{x}}_{k}|{\text{y}}_{1:k-1}\right)=\int p\left({\text{x}}_{k}|{\text{x}}_{k-1}\right)p\left({\text{x}}_{k-1}|{\text{y}}_{k-1}\right)d{\text{x}}_{k-1} \end{array}$$

where *p*(**x**_*k*_|**x**_*k*−1_) models how the system state evolves from current time to the next, and *p*(**x**_*k*−1_|**y**_*k*−1_) corresponds to the a-posterior probability for the previous time instant.

In the update step, the a-posterior prediction is obtained by correcting the a-prior prediction with the information provided by the new measurement data **y**_*k*_:

(2) $$\begin{array}{l} p\left({\text{x}}_{k}|{\text{y}}_{1:k}\right)=\frac{p\left({\text{y}}_{k}|{\text{x}}_{k}\right)p\left({\text{x}}_{k}|{\text{y}}_{1:k-1}\right)}{p\left({\text{y}}_{k}|{\text{y}}_{1:k-1}\right)}=\alpha p\left({\text{y}}_{k}|{\text{x}}_{k}\right)p\left({\text{x}}_{k}|{\text{y}}_{1:k-1}\right) \end{array}$$

where *p*(**y**_*k*_|**x**_*k*_) reflects the mapping between the system states and the measurements, called the likelihood term, and *p*(**y**_*k*_|**y**_1:*k*−1_) is a normalization constant α that can usually be ignored.

Following Equations (1) and (2), *p*(**x**_*k*_|**y**_1:*k*_) can be calculate recursively. In practical implementation, it requires to have closed form solutions to these general equations. Here we explored two specific algorithms, the Kalman filter (Grewal, 2011) and the unscented Kalman filter (UKF) (Julier et al., 1995) for estimating the EMG signals and kinematics recursively from the cortical signals, and details are described as follows.

#### 2.4.2. Kalman filter

For the implementation of the Kalman filter, the state transition and the measurement model were both assumed linear and with Gaussian noise. Supposing model parameters are time invariant, these models can be written as:

(3) $$\begin{array}{rcl} {\text{x}}_{k} & = & \text{A}{\text{x}}_{k-1}+{\text{w}}_{k} \end{array}$$

(4) $$\begin{array}{rcl} {\text{y}}_{k} & = & \text{H}{\text{x}}_{k}+{\text{q}}_{k} \end{array}$$

where **A** is the state transition matrix, **H** is the measurement matrix. For EMG decoding, **A**, **W** ∈ ℝ^*M*×*M*^, **H** ∈ ℝ^*C*×*M*^, and for kinematics decoding, **A**, **W** ∈ ℝ^3×3^, **H** ∈ ℝ^*C*×3^. The random variables ${\text{w}}_{k}\sim N\left(0,\text{W}\right)$ and ${\text{q}}_{k}\sim N\left(0,\text{Q}\right)$ denote the process and the measurement noise respectively. **A** and **H** are fitted from the training data via a least square linear regression approach, while **Q** and **W** are regression residuals. Let ${\hat{\text{x}}}_{k}^{-}$ and ${\hat{\text{x}}}_{k}$ represent the a-prior estimation and the a-posterior estimation of the system state, the error covariance matrices can be defined as

$$\begin{array}{l} {\text{P}}_{k}^{-}=E\left[\left({\text{x}}_{k}-{\hat{\text{x}}}_{k}^{-}\right){\left({\text{x}}_{k}-{\hat{\text{x}}}_{k}^{-}\right)}^{T}\right] \end{array}$$

for the a-prior estimation, and

$$\begin{array}{l} {\text{P}}_{k}=E\left[\left({\text{x}}_{k}-{\hat{\text{x}}}_{k}\right){\left({\text{x}}_{k}-{\hat{\text{x}}}_{k}\right)}^{T}\right] \end{array}$$

for the a-posterior estimation.

The estimation started from a prediction step:

(5) $$\begin{array}{l} {\hat{x}}_{k}^{-}=A{\hat{x}}_{k-1}\\ P_{k}^{-}=AP_{k-1}A^{T}+W \end{array}$$

Subsequently, ${\hat{\text{x}}}_{k}$ was obtained via an update step with the new measurement data at **t**_*k*_:

(6) $$\begin{array}{l} {\hat{x}}_{k}={\hat{x}}_{k}^{-}+K_{k}(y_{k}-H{\hat{x}}_{k}^{-})\\ P_{k}=(I-K_{k}H)P_{k}^{-} \end{array}$$

where ${\text{K}}_{k}={\text{P}}_{k}^{-}{\text{H}}^{T}{\left(\text{h}\text{H}{\text{P}}_{k}^{-}{\text{H}}^{T}+\text{Q}\right)}^{-1}$ is known as the Kalman gain.

#### 2.4.3. ANN based neural tuning model

For the update procedure of recursive Bayesian estimation indicated by Equation (2), knowledge about the mapping from system states to the measurements is required. Here it means a more appropriate model describing the neuronal modulation of muscular activity and limb movements should be selected. Previous studies have indicated that different hierarchically organized components, including the motor cortex and several subcortical structures and also circuits in the spinal cord, are involved in the neural motor control process (Kandel et al., 1995; Harel et al., 2008). Therefore, much nonlinearity exists for the neuronal modulations to movements, which is difficult for modeling with classical parametric techniques. The linear model (4) is actually a straightforward approximation for the neuronal modulations. Although easy for implementation, it may not be consistent with the actual neural system. It is expected to have a model which can reflect the intrinsic nonlinearity of the neural control process.

Here we utilized a feed-forward single hidden layer ANN to construct the model for neuronal modulations to EMG and kinematics. With the ability of approximating any complex nonlinear mappings directly from the input samples, ANN has been widely applied in many fields (Suykens et al., 2012), and is assumed to be efficient for the modeling of the neuronal modulations. The proposed feed-forward network included an input layer, a hidden layer and an output layer. Each neuron in one layer had directed weighted connections to those in the subsequent layer. Sigmoid functions were applied as the activation functions for the hidden layer neurons. Parameters of the network, including the connection weights and activation thresholds, were determined through a learning process. The modeling errors were fed back through the network via a back-propagation mechanism, and a gradient descent method was adopted for parameters adjusting in every learning epoch (Graupe, 2013). The ANN based measurement model can be written as:

(7) $$\begin{array}{l} {\text{y}}_{k}=net\left({\text{x}}_{k}\right) \end{array}$$

where *net*(·) represents the neural network. For EMG decoding, *net*(·):ℝ^*M*^ → ℝ^*C*^ and for kinematics decoding, *net*(·):ℝ^3^ → ℝ^*C*^.

#### 2.4.4. Unscented kalman filter

As an improved algorithm to the Kalman filter, the unscented Kalman filter (UKF) incorporates nonlinear models in estimation, and uses a nonstochastic simulation to approximate the nonlinearity (Julier et al., 1995). The basic procedure for the UKF is the unscented transform, a deterministic sampling technique for approximating a given probability distribution with a series of weighted sample points (Sigma points), which has been proven superior to other ordinary linearization or approximation methods (Wan and Van Der Merwe, 2000).

In our implementation, the system states were still assumed to evolve according to a linear function as Equation (3) in the prediction step, while the mapping from the system states **x**_*k*_ to the measurements **y**_*k*_ was computed according to the ANN based model (7) in the update step.

Let *d* denote the dimension of the state variable. A set of 2*d* + 1 Sigma points were generated from ${\hat{\text{x}}}_{k}^{-}$ and ${\text{P}}_{k}^{-}$ through the unscented transform:

(8) $$\begin{array}{l} X_{k}^{-(0)}={\hat{x}}_{k}^{-}\\ X_{k}^{-(i)}={\hat{x}}_{k}^{-}+\sqrt{d+\lambda }{\left[\sqrt{P_{k}^{-}}\right]}_{i}\\ X_{k}^{-(i+d)}={\hat{x}}_{k}^{-}-\sqrt{d+\lambda }{\left[\sqrt{P_{k}^{-}}\right]}_{i},i=1,2,\ldots ,d \end{array}$$

where [·]_*i*_ denotes the *i*-th row of the matrix, λ = α^2^(*d* + κ) − *d* is a scaling parameter and α and κ determine the spread of the sigma points around ${\hat{\text{x}}}_{k}^{-}$. The square root of the matrix ${\text{P}}_{k}^{-}$ was obtained by calculating the Cholesky decomposition. These Sigma points were propagated through Equation (7) to get the transformed results:

(9) $$\begin{array}{r} {\hat{\text{Y}}}_{k}^{i}=net\left({\text{X}}_{k}^{-\left(i\right)}\right),i=0,1,\ldots ,d,\ldots ,2d \end{array}$$

The predicted mean and covariance of the measurement, and the cross-covariance between the state and the measurement were calculated from these transformed Sigma points:

(10) $$\begin{array}{l} {\hat{Y}}_{k}=\sum_{i\;=\;0}^{2d}w_{i}{\hat{Y}}_{k}^{\left(i\right)}\\ P_{k}^{YY}=\sum_{i\;=\;0}^{2d}w_{i}({\hat{Y}}_{k}^{\left(i\right)}-{\hat{y}}_{k}){\left({\hat{Y}}_{k}^{\left(i\right)}-{\hat{y}}_{k}\right)}^{T}+Q\\ P_{k}^{XY}=\sum_{i\;=\;0}^{2d}w_{i}(X_{k}^{-(i)}-{\hat{x}}_{k}^{-}){\left({\hat{Y}}_{k}^{\left(i\right)}-{\hat{y}}_{k}\right)}^{T}+Q \end{array}$$

where *w*_*i*_ are a series of constant weights:

$$\begin{array}{l} w_{0}=\frac{\lambda }{d+\lambda }\\ w_{i}=\frac{1}{2(d+\lambda )},i=1,2,\ldots ,2d \end{array}$$

The a-posterior estimate ${\hat{\text{x}}}_{k}$ and corresponding error covariance matrix **P**_*k*_ were finally obtained by:

(11) $$\begin{array}{l} {\hat{x}}_{k}={\hat{x}}_{k}^{-}+K_{k}(y_{k}-{\hat{y}}_{k})\\ P_{k}=P_{k}^{-}-K_{k}P_{k}^{YY}K_{k}^{T} \end{array}$$

where the Kalman gain was calculated as ${\text{K}}_{k}={\text{P}}_{k}^{XY}{\left({\text{P}}_{k}^{YY}\right)}^{-1}$. The initial value for the error covariance matrix **P**_0_ was set to the unit matrix. **P**_*k*_ will show a convergent feature as the calculation of Equations (8)–(11) at each time instant.

#### 2.4.5. Performance evaluation

Two metrics, Pearson's correlation coefficient (CC) and the signal to noise ratio (SNR) were used for the performance evaluation of the algorithms.

CC is calculated from the actual signal *x* and the estimated value $\hat{x}$ for each channel by:

$$\begin{array}{l} CC=\frac{\sum_{k=1}^{K}\left(x_{k}-\bar{x}\right)\left({\hat{x}}_{k}-\bar{\hat{x}}\right)}{\sqrt{\sum_{k=1}^{K}{\left(x_{k}-\bar{x}\right)}^{2}}\sqrt{\sum_{k=1}^{K}{\left({\hat{x}}_{k}-\bar{\hat{x}}\right)}^{2}}} \end{array}$$

where *K* is the length of the signal. An CC value close to 1 indicates better fit between the real and estimated data.

SNR is defined as the ratio between the variance of the actual data and the mean square error (MSE) of the estimates, and is calculated as:

$$\begin{array}{l} SNR=10\log_{10}\frac{\sum_{k=1}^{K}{\left(x_{k}-\bar{x}\right)}^{2}}{\sum_{k=1}^{K}{\left({\hat{x}}_{k}-x_{k}\right)}^{2}} \end{array}$$

for each channel. It is quantified in decibels (dB) scale, where 0 dB means the signal and the noise are in equal proportion, and larger positive value means more useful signals are extracted. The SNR is sensitive to the errors introduced by amplitude scaling and offsets, and has also been adopted in Fitzsimmons et al. (2009).

## 3. Results

### 3.1. Synchronized multiple data recordings

**Figure 2:**
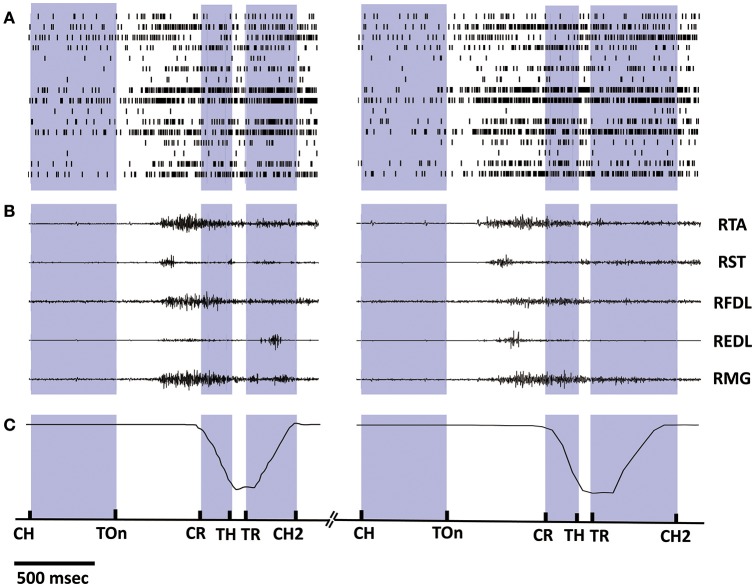
**Synchronized multiple data recorded during 2 successive trials from monkey 1. (A)** Spike trains of 16 cortical neurons. Short bars indicates the actual spiking time and each row corresponds to a neuron. **(B)** EMG signals of 5 lower limb muscles. **(C)** Position trajectories of the marker on the right ankle of the monkey. The exact time instances of important behavior events are indicated on the bottom time line by short bars: Center Hit (CH), Target On (TOn), Center Release (CR), Target Hit (TH), Target Release (TR), Center Hit Again (CH2), and the exact meaning of these events can be found in Section 2.2. Data within the inter-trial interval (2–5 s) are omitted for displaying.

Synchronized multiple data recorded during 2 successive trials from monkey 1 is shown in Figure 2, including the spike trains of 16 task-related neurons (Figure 2A), the raw EMG signals of 5 right lower limb muscles (Figure 2B), and the trajectories of the marker attaching on the monkey's right ankle (Figure 2C). The occurring time instants for certain behavior events are indicated by short bars at the bottom. Data within the inter-trial interval (about 5 s) are omitted for displaying. Notably, the firing rates of most cortical neurons kept low when resting (quiet squatting, Center Hit to Target On). An ensemble of neurons began to discharge more frequently after target presenting (Target On), while their firing rates have been on a significant increase during such pre-movement epoch (Target On to Center Release). Later, amplitude oscillations were observed for some EMG channels around the initiation of the standing (Center Release), indicating the activation of corresponding muscles. For the subsequent epochs involving standing up (Center Release to Target Hit), standing still (Target Hit to Target Release), and squatting down (Target Release to Center Hit Again), some obvious event-related neural discharging patterns can be revealed via a series of peri-event histogram analyses, which have been reported in our previous study (Ma et al., 2015). Compared to the previous work, here we may go a further step to explore the neuron-muscle associations more quantitatively for the neural decoding problems based on these observational or statistical results.

### 3.2. Spatial distributions of different muscle-innervating neurons

In the acute neural recording session, we explored the M1 area in the left hemisphere by changing the locations and penetrating depths of independent movable electrodes at a distance of 1 mm. The exact range of the region was in the coordinate system determined by the stereotaxic apparatus: 8.00–22.00 mm from anterior to posterior (PA), 0.00–16.00 mm from dorsal to lateral (DL), and 0.00–5.00 mm in the depth. A total number of 96 datasets were collected, and 1598 task-related neurons were isolated (909 from Monkey 1, 689 from Monkey 2).

**Figure 3:**
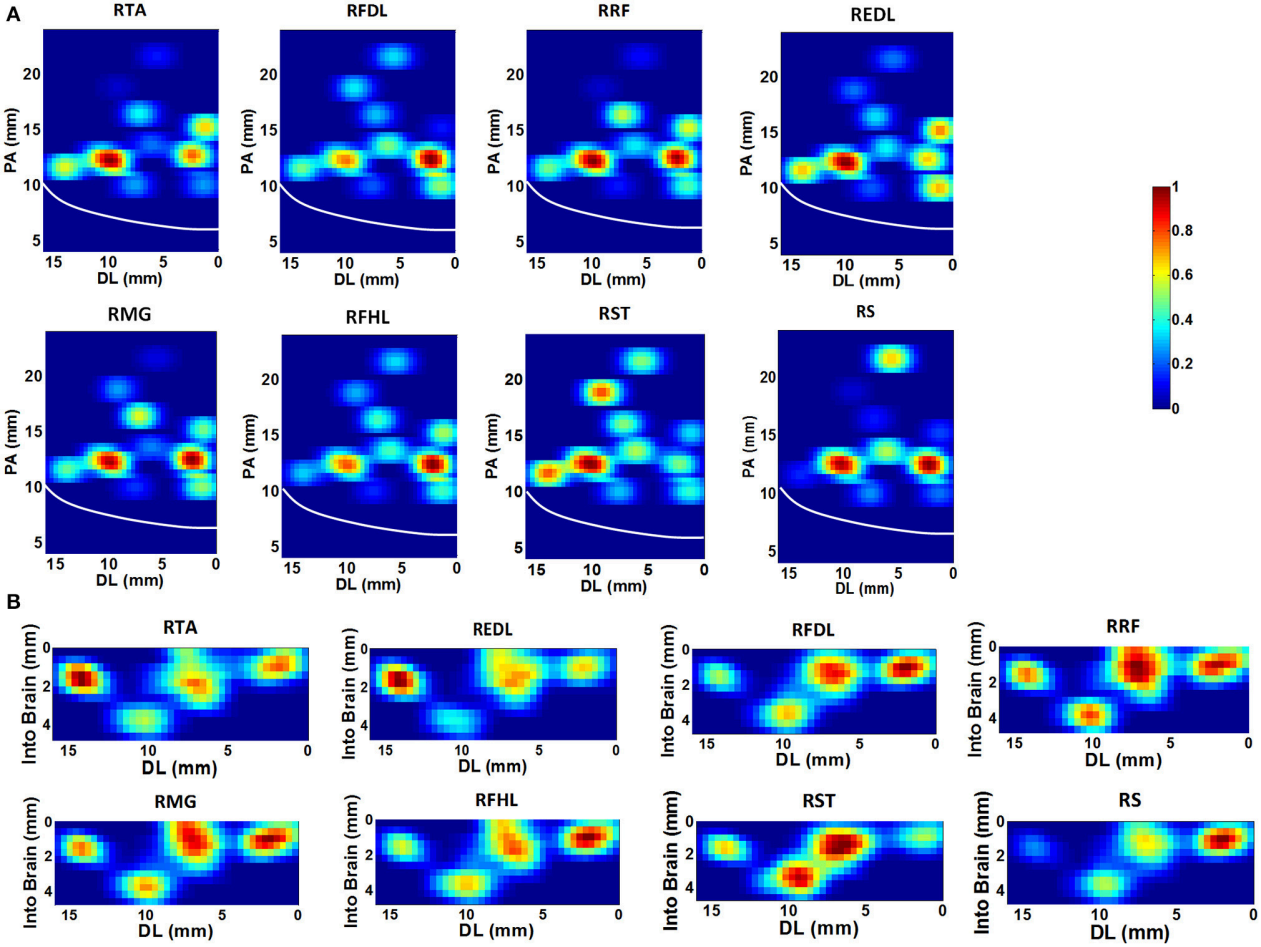
**The density contours of the identified neurons with regard to 8 muscles, generated with the data from monkey 1**. The density values were normalized to [0,1], and smoothed with a median filter, quantified by the color-bar. **(A)** Horizontal view. The x-axis represents the Dorsal-Lateral (DL) dimension and y the Posterior-Anterior (PA) dimension. Each grid represents a 0.4 × 0.4 mm area. The white line indicates the position of the central sulcus. **(B)** Coronal view. The x-axis still represents the DL dimension and the y-axis indicates the depth. The resolution of each grid is 0.4 × 0.4 mm.

Based on the SpTA analyses mentioned above, the neurons which may facilitate the activation of each given muscle were identified from the ensemble. Figure 3 shows the density contours of the identified neurons with regard to 8 muscles in the horizontal (Figure 3A) and the coronal (Figure 3B) view using the data from monkey 1. The central sulcus (CS), as a prominent landmark, is also indicated designedly in the plots. All density values are normalized to [0, 1] and smoothed by a median filter. The hotter color indicates the higher density. These plots are to some extent “maps” for finding the neurons which may contribute to improving the accuracy of the EMG decoding. In the horizontal view, several concentration centers of muscle-innervating neurons for the 8 muscles can be clearly identified (Figure 3A). In the coronal view, we noticed that the depth between 0.5 ~2.5 mm was where muscle-innervating neurons aggregated most densely (Figure 3B).

**Figure 4:**
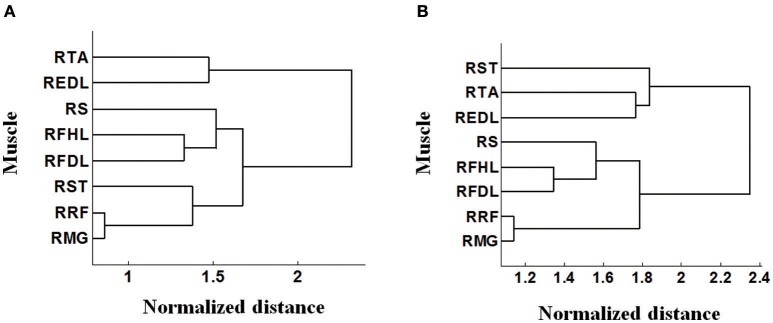
**Cluster analysis based on the pattern of density contours of different muscle-innervating neurons corresponding to each given muscle. (A)** Clustering results on the horizontal view (DL-PA). **(B)** Clustering results on the coronal view (DL-depth).

It is notable that some density contours are quite analogous, like those for the RRF-innervating neurons and the RTA-innervating neurons. But clear divergence exists for some other groups, like the RRF and the REDL. Here we evaluated the similarities of the spatial distributions of different muscle-innervating neurons via a distance measure based cluster method. To be specific, the density contours for different types of muscle-innervating neurons can be viewed essentially a series of matrices. The Euclidean distances between every two matrices were calculated for a cluster analysis. The results of the cluster are shown in Figure 4, for the horizontal (Figure 4A) and the coronal view (Figure 4B) respectively, using the data from Monkey 1. Several groups of muscles could be identified from the cluster results: RRF and RMG, RFHL and RFDL, RTA and REDL, either in the horizontal or the coronal view. Dramatically, the results using the data from Monkey 2 are almost the same. We seek possible illustrations for these results with some knowledge in anatomy. In fact, RMG and RRF are both thigh muscles. RFHL and RFDL are leg muscles, and are both innervated by the Tibial Nerve. RTA and REDL are also leg muscles, but are innervated by the Deep Fibular Nerve (Moore et al., 2011). These results suggest that, for muscles sharing larger similarities in anatomy, the corresponding M1 neurons have closer spatial distributions.

### 3.3. EMG decoding

The chronic neural recording session lasted 2 weeks and 13 datasets were collected for each monkey. In each dataset, 40 ± 5 successful stand/squat trials were recorded, and an average of 34 task-related neurons can be identified from all detected units. A 4-fold cross-validation was employed for model parameter estimation, in which 3-folds were used for training and 1-fold for testing.

**Figure 5:**
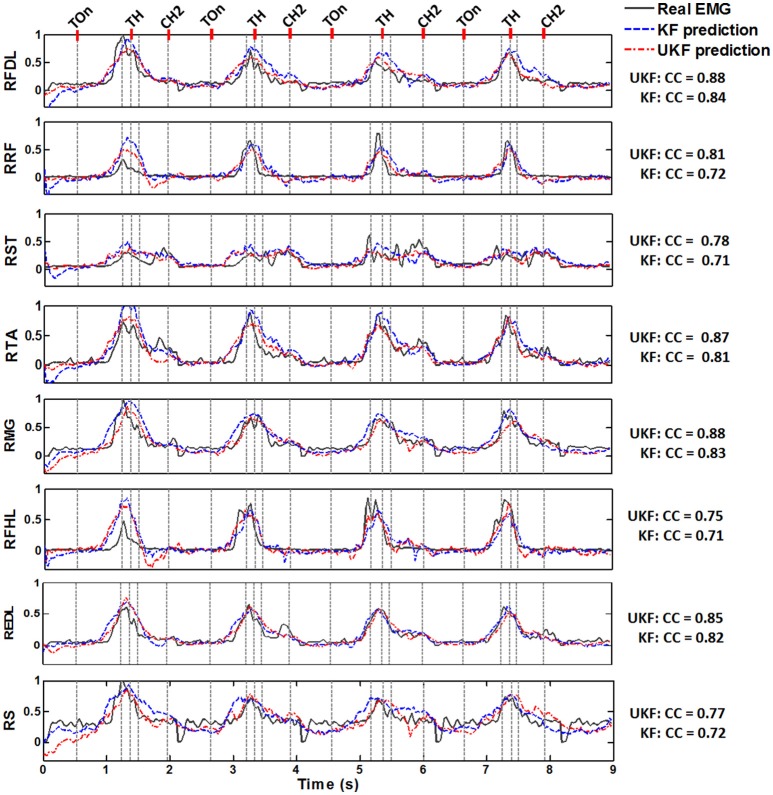
**Normalized actual EMG envelop waveforms (dark gray solid) and the reconstructed results by the standard Kalman filter (KF, blue dashed) and the UKF (red dash dot) in four successive stand/squat trials**. The data are from one of the chronic datasets from Monkey 1. The subplot in each row corresponds to a muscle, where the name is labeled on the left. The occurring time of 5 important behavior events are indicated by a series of light gray dashed lines in each subplot, where the names of 3 events could be found on the top line of the figure: Target On (TOn), Target Hit (TH), and Center Hit Again (CH2). The event occurring right before Target Hit is Center Release, and the event right after Target Hit is Target Release. The exact meaning of the behavior events can be found in Section 2.2. The CC between the actual and predicted signals are listed on the right of each subplot for the two algorithms.

Figure 5 shows typical plots of the predicted EMG envelops(blue dashed line, by the standard Kalman filter; red dash dot line, by the UKF) from a test subset in comparison with the actual EMG signals(dark gray solid line) during 4 successive trials conducted by Monkey 1. The amplitude of all signals are normalized to the interval [0, 1]. The exact time of 5 important behavior events are indicated in each subplot (light gray dashed line). It is perceived from the plots that both of the proposed decoding methods were capable to accurately predict the overall trends of the EMG signals during the standing and squatting task, as well as some significant waveform features such as the quiescence before Target On, the gradual amplitude increase during the pre-movement epoch (Target On to Center Release), and the transient changes during the movement stages. Compared to the standard Kalman filter, the UKF method could produce more approximate EMG envelop values. These results clearly imply that not only the timings for muscle activation/relaxation, but also the strength of lower limb muscular activity can be extracted from the cortical ensemble signals.

**Figure 6:**
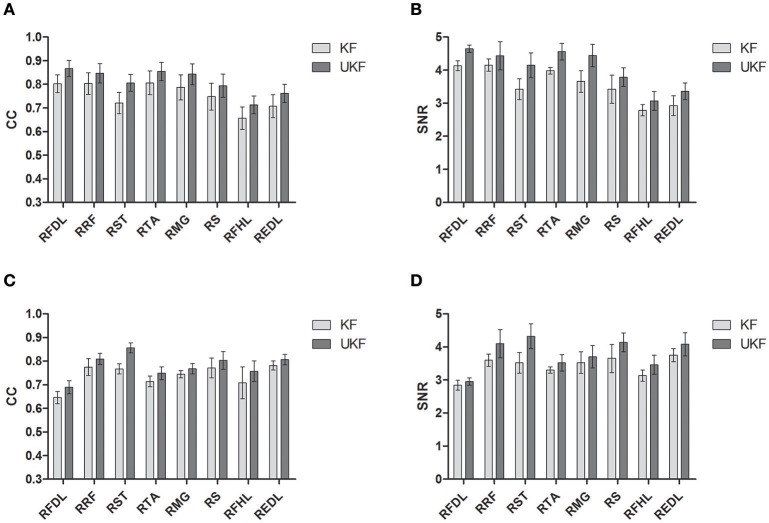
**The CC and SNR for the EMG decoding**. The mean and the standard error of the mean (SEM) are shown. The SEM is indicated by the error bar. **(A,B)** are for Monkey 1. **(C,D)** are for Monkey 2.

Figure 6 shows the CC and SNR between the predicted and real EMG signals in each channel for the 2 monkeys, demonstrating the mean and the standard error of the mean (SEM). It is suggested that the UKF based decoding method can achieve higher CC and SNR values. For Monkey 1, the mean CC values for 7 channels of EMG signals (RFDL, RRF, RST, RTA, RMG, RS, REDL) were greater than 0.75, and the mean SNR values for 6 channels (RFDL, RRF, RST, RTA, RMG, RS) exceeded 3.5, when using the UKF as the decoding algorithm. In contrast, when using the standard Kalman filter to predict the EMG signals, there were only 4 channels (RFDL, RRF, RTA, and RMG) of which the mean CC values exceeded 0.75, and only 3 channels (RRF, RTA, and RMG) of which the mean SNR value was greater than 3.5. Similar results can be perceived for Monkey 2. Afterwards, we performed a right tail *t*-test with 416 observations (13 datasets, 4-folds, 8 channels of EMG signals) and significance level α = 0.05 for each monkey to test whether the UKF based method improved the EMG decoding performance significantly. The *t*-test verified that the predicted signals with the UKF had higher CC and SNR values compared to those with the standard Kalman filter for all EMG channels (*p* < 0.001). In fact, the UKF improved the CC for 13.6% and the SNR for 17.5%.

### 3.4. Kinematics decoding

**Figure 7:**
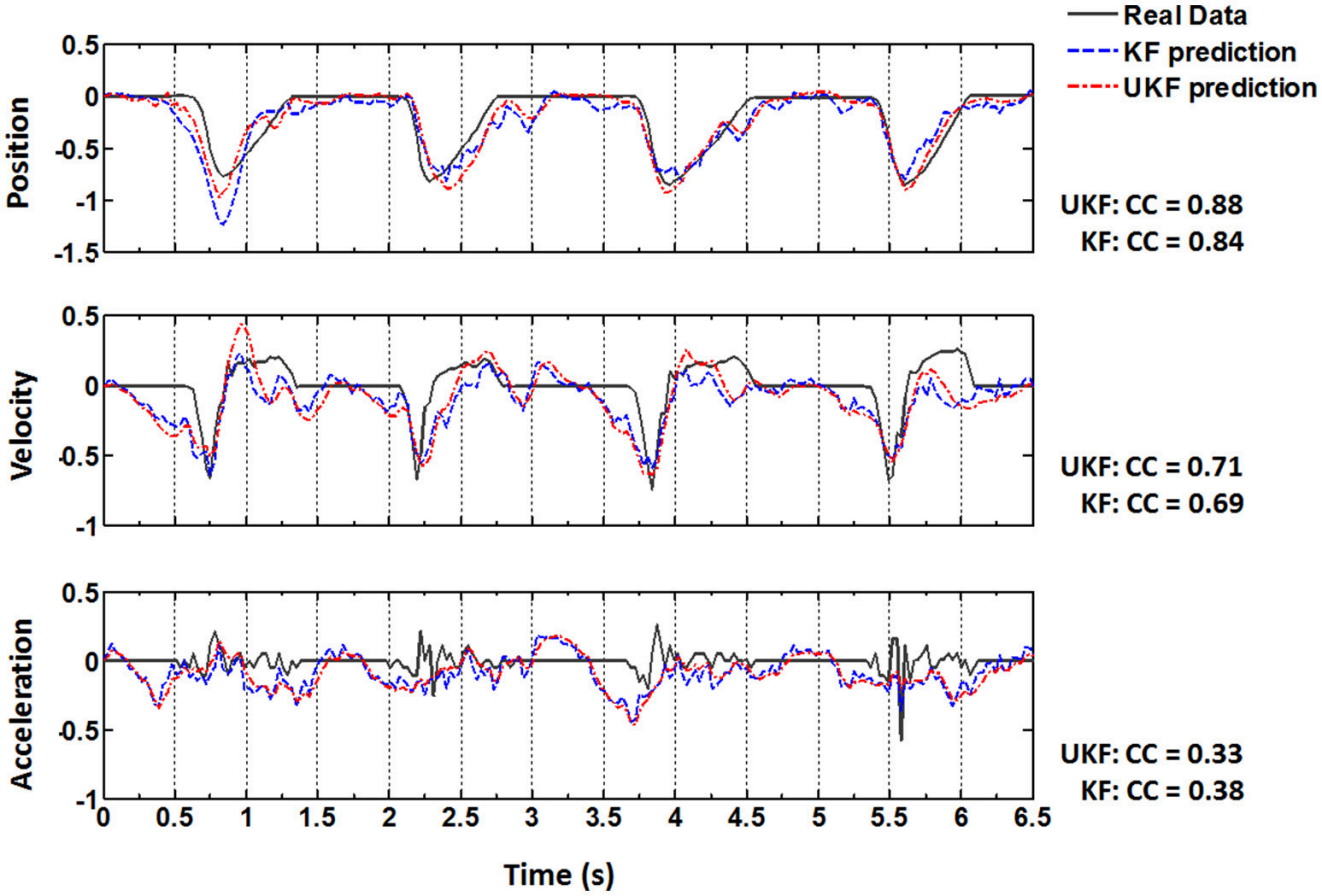
**Real position, velocity and acceleration (dark gray solid) and the reconstructed results by the standard Kalman filter (blue dashed, KF) and the UKF (red dash dot) in four successive trials**. The CC values between the actual and reconstructed trajectories are listed on the right for each subplot.

The same procedures for model training and testing as in EMG decoding were adopted for the kinematics decoding. Figure 7 presents the predicted trajectories for the position, velocity and acceleration of the ankle marker in comparison with the real data (dark gray solid line). It is shown that both methods can provide reliable tracking for the trajectories of the position when the legs of the monkey were moving either downward or upward. For the velocity, the predictions can approximate the overall trend but discrepancies were found within some time intervals. However, both methods cannot achieve satisfactory predictions for the acceleration. Table 1 summarizes the CC and SNR between the predicted and real trajectories (mean ± SEM). For tracking the position, high mean CC and SNR values were achieved. A right tail *t*-test with 52 observations (13 datasets, 4-folds) and significance level α = 0.05 for each monkey verified the superiority of the UKF based method (*p* < 0.001). For approximating the velocity, the CC and SNR values were relatively low, but higher than 0.5. For decoding the acceleration, both algorithms encountered not good enough performance, with the mean of the CC < 0.5, and SNR < 1.

**Table 1:** **The CC and SNR values between the predicted and actual data for kinematics decoding (mean ± SEM)**.

		**Monkey 1**		**Monkey 2**	
		**CC**	**SNR(dB)**	**CC**	**SNR(dB)**
Prediction using	Position	0.85 ± 0.05	5.53 ± 0.44	0.83 ± 0.05	5.05 ± 0.52
the standard	Velocity	0.61 ± 0.06	1.58 ± 0.75	0.64 ± 0.05	1.67 ± 0.84
Kalman filter	Acceleration	0.37 ± 0.05	0.55 ± 0.34	0.33 ± 0.03	0.45 ± 0.33
Prediction using	Position	0.90 ± 0.05	6.12 ± 0.83	0.88 ± 0.05	5.93 ± 0.74
the UKF	Velocity	0.66 ± 0.08	1.89 ± 0.95	0.68 ± 0.08	1.52 ± 1.17
	Acceleration	0.36 ± 0.10	0.53 ± 0.58	0.34 ± 0.08	0.47 ± 0.27

### 3.5. Stability analysis of the neural decoding algorithms

**Figure 8:**
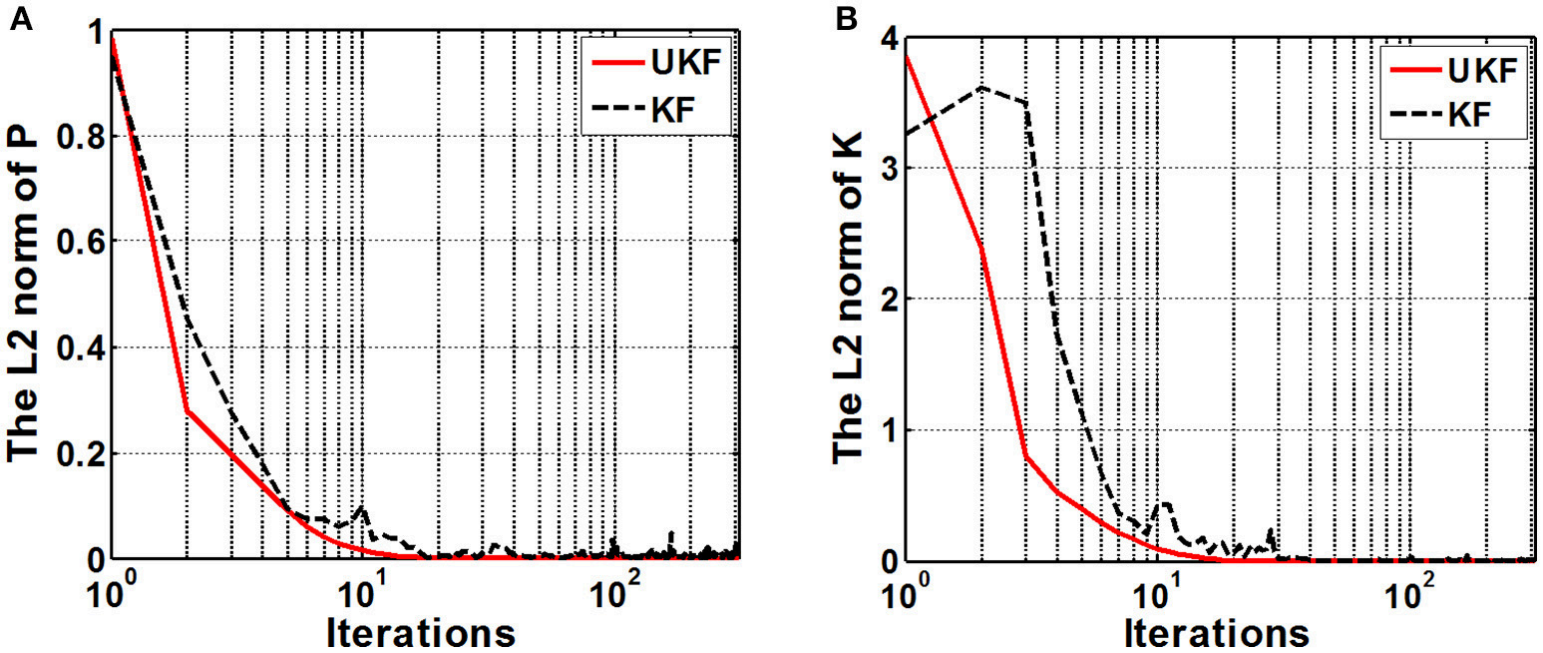
**The *L*^2^ norm of the a-posteriori estimate error covariance matrix P_*k*_ (A)** and the Kalman gain matrix **K**_*k*_ **(B)**. The x-axis indicates the iterations (*k*) and is expressed in logarithm form. Red solid line and black dashed line represent the UKF and the standard Kalman filter situation respectively.

We examined the stability of the neural decoding algorithms by investigating the convergence of the a-posteriori estimate error covariance matrix **P**_*k*_ and the Kalman gain **K**_*k*_. The matrix **P**_*k*_ defines the estimation error at each time step, while **K**_*k*_ represents the relative importance of the measurement residual with respect to the a-prior estimation ${\hat{\text{x}}}_{k}^{-}$. For reliable estimations, it is expected that the errors converge to considerable small values as the recursive iteration increases. As **P**_*k*_ gets small, it means there is no requirement to alter the estimation too much at each time instant, therefore, **K**_*k*_ will also get small. So the numerical stability of **P**_*k*_ and **K**_*k*_ are appropriate indexes for evaluating the stability of the neural decoding algorithms. Figure 8 plots the *L*^2^ norm of **P**_*k*_ and **K**_*k*_ across iterations. It is shown that the *L*^2^ norm of both **P**_*k*_ and **K**_*k*_ converge to a small value after about 20–30 iterations. It is also suggested that the UKF can achieve faster convergence rate for **P**_*k*_ and **K**_*k*_.

## 4. Discussion

In this study, we demonstrate feasible approaches for decoding lower limb muscular activity and kinematics from cortical neural signals during monkey performing a series of visual cued stand/squat tasks in a virtual environment. This study provides important insight regarding the cortical innervation of voluntary lower limb movements, while previous work have paid ample attentions to the upper limbs. The results clearly demonstrate that lower limb EMG signals and kinematics can be predicted from the firing rate sequences of multiple cortical neurons, which suggests the possibility for more comprehensive lower limb BMI systems.

The work in Bulea et al. (2014) has demonstrated sitting and standing intention can be discriminated from scalp EEG signals. However, more details about the timing and strength of multiple muscle activity and kinematics are still difficult to be extracted from the general noninvasive signals. There are not too many literatures concerning lower limb motor control using invasive methods due to the lack of systematic experimental protocols like Center-Out in upper limb circumstance and the uncertainty about brain areas for electrode implantation. Similar to the work in Fitzsimmons et al. (2009), we performed invasive neural recordings, but we concentrated voluntary stand/squat instead of the cyclic bipedal walking. By exploring a wide region in M1 with movable electrodes, we have identified the spatial distributions of different muscle-innervating neurons corresponding to each given muscle, which can provide important guidance for implanting chronic electrodes. The recursive Bayesian estimation based methods proposed here are proved effective for implementation, and could produce accurate EMG and kinematic estimation.

In Pohlmeyer et al. (2007), the prediction of EMG signals of several arm and hand muscles in button pressing or grasp movements captured most EMG features, and got *R*^2^ values between 0.60~0.70. In Li et al. (2009), the moving trajectories of the hand of the monkey can be reconstructed off-line with SNR between 2 and 5 dB, or CC between 0.6 and 0.9. In our study, the neural decoding accuracies of the 8 lower limb muscles and the ankle marker position are comparable to these studies (Figure 6, Table 1). Such results suggest that current upper limb BMI design concepts can be extended to lower limb occasions.

### 4.1. Implications for the cortical neural encodings of voluntary lower limb movements

Previous upper limb BMI studies found neurons in M1 (Moran and Schwartz, 1999; Paninski et al., 2004; Wahnoun et al., 2006; Truccolo et al., 2008), ventral premotor cortex (Prabhu et al., 2009; Carpaneto et al., 2011), dorsal premotor cortex (Hao et al., 2013) encode various task related parameters, like reaching direction, joint orientation, or hand gestures, and have developed different neural decoding algorithms based on such observations (Hatsopoulos et al., 2004; Hao et al., 2013, 2014). As indicated in Winters and Woo (2012), motor control process of lower limbs is often considered different from that of upper limbs. We wonder either abstract representation of movements or explicit encodings of muscular activity and limb states can be extracted from the neural responses in M1 lower limb area, which determines the feasibility for constructing lower limb BMI systems.

Our findings suggest M1 neural ensemble may provide specific encodings to the execution of such visual guided standing/squatting task. When the visual cue was launched at Target On, the animal commenced to plan its movement in order to get success (Figure 1B). At this moment, the firing rates of a large portion of neurons have begun to increase. After the movement was initiated (at Center Hit), a good control about the position of the legs must be maintained since the monkey was required to match the ankle marker to the figures on the screen in right sequence, while most neurons were observed keeping relatively high firing rate. We think this fact reflects the sustained activity of task-related neurons from planning to executing in voluntary movement. In contrast, most recorded M1 neurons showed high variant firing patterns and exhibited weak modulation to the movement in a treadmill bipedal walking task (Huo et al., 2010). The execution of walking can be attributed to sort of cyclic movements, during which low level centers, like the CPG, take over more control details. Thus, the synchronization of M1 neural activity were absent. However, also in Huo et al. (2010), almost all recorded neurons became increasingly synchronized in firing patterns to each step, especially before and during stance phase, when the monkey walked on the treadmill after a spinal cord hemi-section surgery. These observations imply that M1 neurons are intensively involved into the control of walking when spinal cord is impaired. For the visual guided standing/squatting tasks, accurate responses to certain cues must be produced in time, of which the complexity is far more than the cyclic walking, and beyond the spinal cord circuits can handle. To sum up, M1 neurons participate the specific control of lower limb movements more in those circumstances during which low level centers are damaged or unable to produce accurate responses for purposeful tasks. Compared to another bipedal walking study (Fitzsimmons et al., 2009), the SNR for the predictions of EMG signals on the right leg musculature was 1.55 ± 0.39, which was lower than ours (Figure 6). This fact indicates the neural activity patterns during the visual cued standing/squatting is sufficient for achieving better decoding performance than the cyclic walking.

### 4.2. Functional division of M1 neurons corresponding to muscles

To determine the best locations for chronic electrode implantation, we investigated the contribution of a number of M1 neurons to facilitate given muscles. In fact, the elaborate organization of M1 neurons in lower limb control process can be revealed by plotting the density contours of neurons corresponding to each given muscle (Figure 3). The detected neurons appear sort of functional division corresponding to muscles or muscle groups. On one hand, there are significant discrepancies among the spatial distributions of different muscle-innervating neurons. On the other hand, analogous or overlapping spatial distributions of neurons can be identified for muscles innervated by the same nerve (Figure 4). The spatial range of the lower limb representative area in M1 has been repeatedly confirmed in the last decades using imaging techniques such as functional magnetic resonance imaging (fMRI), but the exact muscle-neuron associations and the specific locations of different muscle-innervating neurons are still not clear. Compared to these methods, the simultaneous multiple data recordings in this study achieved better time and spatial resolutions. Since the actual number of neurons is huge, we cannot yet declare we have constructed an exact map between M1 neurons and lower limb muscles. The most direct benefit is that we are more clear about which locations are optimal for the chronic electrode implantation. Meanwhile, these findings are helpful for understanding the organizations of the motor control pathway for lower limbs.

### 4.3. Nonlinear method achieves better decoding performance

We try to seek better computational models to relate the multi-dimensional neural firing rate sequence to the EMG signals or kinematics. It is noticeable that higher decoding accuracy was achieved when using the UKF with an ANN based neural tuning model to estimate the EMG signals and the trajectories of the ankle position. Considering the intrinsic nonlinearity in the neural system as described before, a linear model as 4 may not fit the real neural data very well, though it is easy for implementation. The superiority of incorporating nonlinear models for neural decoding has been revealed by some previous studies. In Li et al. (2009), a quadratic neural tuning model relating position, velocity with the binned neural firing rates can considerably improve the decoding accuracy of the 2D hand moving trajectory. In Pohlmeyer et al. (2007), the *R*^2^ between the real and predicted upper limb EMG signals was 0.69 when using linear estimation method, and increased to 0.75 when using nonlinear estimation method. The results in these reports are consistent with ours as mentioned above. The ANN reflected the nonlinearities between the system states to be estimated and the observed multi-dimensional neural firing rate sequences, and improved the decoding accuracy significantly when it was incorporated as the measurement model in the recursive estimation process.

### 4.4. EMG decoding vs. kinematics decoding

We notice that the decoding accuracies for the EMG signals and kinematics are different. As shown in Figure 6, the mean CC values for the 8 channels of decoded EMG signals are all above 0.6, and the mean SNR values are all above 2 dB. For the trajectories of the ankle marker position, the prediction is accurate (mean SNR > 5 and mean *CC* > 0.75). For velocity, the prediction captures the overall trend (SNR around 2 and CC around 0.6). However, the estimation of the acceleration is not satisfactory. In fact, the same situation have been noticed in previous studies. In Wu et al. (2002), the reconstruction for hand position and velocity are satisfactory while for acceleration is poor. They thought the acceleration was a second order difference of position, thus the measurements of it tended to be very noisy in real data. They also indicated the acceleration has a weak effect on the model relative to position and velocity.

After Target On, the monkey was in a plan stage, but actual movement has not been initiated until Center Release. The actual position, velocity and acceleration during the plan stage retained still (Figure 7, real data). Although the overall prediction accuracy for the ankle position was very high, the estimated values often failed to capture such quiescence (Figure 7, upper panel, predicted data). However, many neurons have begun to fire intensively and the amplitudes of the EMG signals have shown an analogous activated profile at the same time (Figure 7). In contrast to the ankle position, the estimated EMG values in this stage captured the dramatic increase of the real signals. Generally speaking, EMG signals are stochastic and noisy, and have greater bandwidth, which suggests more difficulties for the neural decoding algorithms. So the mean CC and SNR for the estimated ankle position trajectories are higher than those for the estimated EMG signals. But compared to the kinematics, EMG signals are closer to the cortical neural activity. When initiating and maintaining a movement, M1 neurons modulate their firing patterns to facilitate the contractile force in the skeletal muscles via a hierarchic downstream pathway. The planned movement are produced by the synergic activity of multiple muscles, and is more indirect with the cortical neurons. So the estimated EMG signals from the cortical activity can capture the trend of the real signals well even in the pre-movement epoch.

### 4.5. Potential applications for FES and mind controlled prostheses

The BMI technology constructs a bridge between the subject's mind and the external actuators. It has been assumed as the most promising technique for neuro-prostheses control and neuro-rehabilitation implementation. Here we expect the findings in our explorations on monkey stand/squat behaviors could be helpful for transforming BMI design concepts to potential clinical applications.

One of the possible applications is using the predicted EMG signals as the inputs of the functional electric stimulation (FES) to activate specific lower limb muscles of the paralyzed patients. FES is an effective method for preventing the muscle mass loss and promoting the functional recovery after nervous system lesion. In Moritz et al. (2008), a monkey can directly control stimulation of muscles using the activity of neurons in M1, thereby restoring goal-directed movements to a transiently paralyzed arm. Here we have demonstrated the timing and strength of the activity of multiple lower limb muscles can be predicted from cortical neural spike trains. With the predicted data, the timing and intensity of the FES for target muscles can be tuned according to the subject's mind to conduct more purposeful stimulation. Such approaches are expected to achieve similar results as in Moritz et al. (2008) for restoring various voluntary lower limb movements, like standing and squatting.

The predicted lower limb kinematics could be incorporated as the control source to drive artificial legs, exoskeleton or walking assistant robots designed for lower limb paralyzed patients or amyotrophic lateral sclerosis (ALS) patients. The actuators can be controlled to track the estimations of position and velocity extracted from cortical neural signals. Compared to traditional methods using accelerometer or other sensors (Reza et al., 2014), brain control enables the patients to manipulate complex biomechatronic devices in a more harmonized manner.

Meanwhile, the proposed algorithms are easy for implementation in micro-chip or real time embedded system. The Kalman filters does not require long windows to collect data as the multiple linear regression method and provides an explicit generative model, which is propitious to real time implementations for capturing rapid motions. The realization of the ANN and the unscented transform have also been proven feasible on current programmable micro controllers.

## 5. Conclusions

In this study, we focus on neural signals collected during monkey performing visual guided stand/squat tasks. We have demonstrated lower limb EMG signals and ankle moving kinematics during stand/squat could be accurately decoded from a group of M1 neurons with the proposed algorithms. Our findings provide new insights for extending current BMI design concepts on upper limbs to lower limb circumstances. Brain controlled exoskeleton, prostheses or neuromuscular electrical stimulators for lower limbs are expected to be developed for the future extension of this work.

## Author contributions

Conceived the project and designed the technical detail: JH (Jiping He). Performed the experiments: CM, JX. Analyzed the data: XM, CM, PZ, JH (Jian Huang). Drafted the manuscript: XM, CM. Revised the manuscript: XM, CM, JH (Jian Huang), JH (Jiping He).

## Funding

This work is supported by the National Natural Science Foundation of China (31460263, 61233015), 973 Project of China (2013CB329506), and Jiangxi Natural Science Foundation (12004630).

### Conflict of interest statement

The authors declare that the research was conducted in the absence of any commercial or financial relationships that could be construed as a potential conflict of interest. The reviewer GL and handling Editor declared their shared affiliation, and the handling Editor states that the process nevertheless met the standards of a fair and objective review.
